# Supplementary material for: Effects of Low Dietary Cation and Anion Difference on Blood Gas, Renal Electrolyte, and Acid Excretions in Goats in Tropical Conditions
Source: Animals (Basel). 2022 Dec 6;12(23):3444. doi: 10.3390/ani12233444 (PMC9740077; doi:10.3390/ani12233444)
Supplement: Supplementary file 1 [file animals-12-03444-s001.zip › animals-2004546-supplementary.pdf]

Supplementary Materials: Effects of low dietary cation and anion difference regimen blood gas and renal electrolyte excretion in goats under tropical conditions

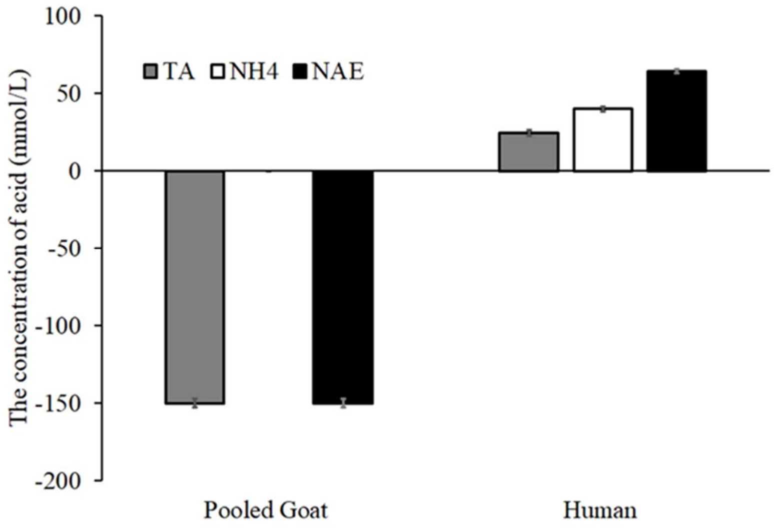

Figure S1. Human urine samples were used as controls for the analysis of acid excretion. Pooled goat urine samples were used to represent samples from goat urine. The analysis procedure was similar to that for goat urine, as described in the Materials and Methods section. The titratable acid (TA), ammonium (NH_4), and net acid excretion from human urine were similar to those previously reported, 24.5 ± 2.0, 39.8 ± 1.7, and 64.3 ± 1.1 mmol/L, respectively (Chan, 1972). The TA, NH_4, and NAC values from the pooled goat urine were -150.0 ± 2.8, 0.0, and -150.0 ± 2.8 mmol/L, respectively.
